# The relative importance of family socioeconomic status and school-based peer hierarchies for morning cortisol in youth: An exporatory study

**DOI:** 10.1016/j.socscimed.2009.12.006

**Published:** 2010-04

**Authors:** Patrick West, Helen Sweeting, Robert Young, Shona Kelly

**Affiliations:** a MRC Social & Public Health Sciences Unit, 4, Lilybank Gardens, Glasgow G12 8RZ, UK; b Centre for Intergenerational Health Research, University of South Australia, Division of Health Sciences, Social Epidemiology Unit, P4-18, City East Campus, North Terrace, Adelaide, SA 5001, Australia

**Keywords:** UK, Youth, Health inequalities, Social hierarchies, Socioeconomic status (SES), School peer group, Stress, Cortisol, Psychoneuroendocrine response, Scotland

## Abstract

This paper examines the relative importance of family socioeconomic status (SES) and school-based peer hierarchies for young people's psychoneuroendocrine response, represented by cortisol level. Data are drawn from a study of 2824, 15-year-olds in 22 Scottish secondary schools in 2006 who provided information on family SES (parental occupation, material deprivation and family affluence) and social position in school hierarchies, together with two morning salivary cortisol samples. School social position was assessed by participants placing themselves on seven ‘ladders’, from which three factors were derived, termed scholastic, peer and sports hierarchies. Controlling for confounds, there was little or no variation in cortisol by any SES measure. By contrast, each school hierarchy was independently associated with cortisol, but in different ways. For the scholastic hierarchy, an inverse linear relationship was found for females, cortisol increasing with lower position. For peer hierarchy, an opposite (direct) linear relationship occurred for males, while for females elevated cortisol was associated only with ‘top’ position. For sports, elevated cortisol among males was associated with ‘bottom’ position, among females with all except the ‘top’. These results are interpreted in the context of Sapolsky's (Sapolsky, 2005) predictions for stress responses to hierarchical position in stable and unstable social systems, the former represented by the scholastic hierarchy involving elevated cortisol in lower positions, the latter by peer hierarchy with elevated cortisol in higher positions. Overall, the results highlight the greater importance of school-based peer groups than family SES for young people's psychoneuroendocrine response.

## Introduction

All social systems are characterised by social hierarchies, a characteristic that applies as much to institutions within societies as it does to whole societies (Marmot, 2004). Whether based on differences in wealth, power, status, employment grade or simply popularity, an individual's position in a hierarchy is likely both to reflect, and have consequences for, a wide range of individual attributes. Most importantly this includes health, one hypothesized mechanism linking social position to health being ‘stress’, or more precisely the psychoneuroendocrine response (PSR) and subsequent impact on physiological processes. This paper focuses on the social hierarchies of young people, with the aim of assessing the relative importance of school-based peer hierarchies and family socioeconomic status for PSR, here represented by cortisol.

### The broader context: Health inequalities, psychosocial mechanisms and the PSR

In society as a whole, an individual's position in the social hierarchy is typically represented by socioeconomic status (SES) measured by various indicators such as income, deprivation and social class. There is now a substantial evidence-base demonstrating that SES in adulthood is systematically related to both physical and mental health (Demakakos, Nazroo, Breeze, & Marmot, 2008), those at the bottom of the social hierarchy experiencing poorest health, those at the top the best. Irrespective of SES measure, the relationship typically takes the form of a social gradient, a phenomenon not compatible with a simple materialist explanation (Macintyre, 1997).

While the causes of ‘health inequalities’ remain a matter of debate, recent work has emphasized psychosocial explanations, and particularly the role of ‘stress’ variously defined as differential exposure to cumulative environmental stressors (McEwen, 1998), perceived lack of control (Kunz-Ebrecht, Kirschbaum, & Steptoe, 2004) or negative feelings associated with unfavourable social comparisons (Wilkinson, 1996). This emphasis owes much to research on non-human primates, especially Sapolsky's (2005) work which has shown how cortisol is affected by an animal's position in the social hierarchy. The evidence shows different effects depending on the stability of the social hierarchy, more stable systems generally conferring advantages to dominant animals (lower cortisol) and disadvantages to subordinates (higher cortisol). By contrast, in unstable hierarchies dominant individuals lose the advantage of high status and are exposed to particularly high levels of competition or challenge resulting in heightened PSR. It is notable that in this work, disadvantage is reflected in elevated cortisol levels.

While the ‘stress’ explanation appears to fit in the case of adult health in stable societies, it is less obvious how it fits with that relating to the SES patterning of health in youth. At this stage in the life-course, when position in the wider social hierarchy is ascribed by family SES, most studies find little or no SES variation in a range of subjective health indicators (e.g. West & Sweeting, 2004), a pattern sometimes referred to as ’relative equality' (West, 1997). However, there is some evidence of variation by SES measure, a few studies finding stronger relationships with ‘family affluence’ (FAS) than parental social class (Holstein, Parry-Langdon, Zambon, Currie, & Roberts, 2004). Developed as an alternative indicator of material dimensions of SES (Currie et al., 2008), FAS is comprised of consumables (e.g. cars, home computers, holidays), which are visible indicators of a family's position in the SES hierarchy. Inasmuch as this generates negative social comparisons, FAS might be more strongly related to the PSR than other SES measures.

### Social hierarchies in youth

The lack of relationship between family SES and health raises questions about the salience of the SES hierarchy as a source of stress in youth and directs attention to other social institutions in which young people are located. Chief among these is the school, which is quintessentially hierarchical in nature. Within any school, pupils are differentiated by school year, ability and academic achievement, either formally in groups or by individual test results or grades. Schools also have a regulative purpose, differentially rewarding pupils for ‘good’ behaviour. In combination with academic success, this defines what makes a ‘good’ pupil, placing them on a hierarchy we have termed ‘scholastic’ (Sweeting, West, Young, & Kelly, submitted for publication). Furthermore, in many societies, schools are the source of officially sanctioned extra-curricular activities, a notable example of which is sport. On the assumption of stability, it might be expected that a pupil's position in the ‘official’ school hierarchy (e.g. academic or sports success) would be inversely associated with the PSR, lower positions incurring greater ‘stress’.

The school, however, is not simply comprised of a single ‘official’ hierarchy but constitutes an arena within which peer group structures and related hierarchies are developed. Such hierarchies refer to a range of attributes with particular salience for young people as desirable youth identities, typically involving judgments about physical appearance, body shape, clothing and style. A voluminous literature testifies to the important role such attributes play as signifiers of group membership, youth subculture, and position in the peer social hierarchy (Milner, 2006). The evidence also shows this is particularly important in mid-adolescence when peer group activity is at a maximum (Giordano, 2003), and popularity in males is generally associated with physical prowess and sports success, in females with attractiveness and spending power (Meisinger, Blake, Lease, Palardy, & Olejnik, 2007). While most research has focused on those who occupy low status in the peer group, and who are most likely to be exposed to ‘stress’, more recent studies have focused on the top of the peer hierarchy and on different dimensions of popularity (Cillessen & Rose, 2005). The precariousness of top positions is indicated in one study which found ‘top girls’ not only experienced, but were perceived as experiencing, considerable pressure to maintain their high status identity as attractive, cool and popular (Michell & Amos, 1997). This association of higher position with negative consequences is similar to that described by Sapolsky for unstable social systems, and may characterize some peer generated hierarchies in youth.

School-based peer groups are, therefore, unlikely to be unidimensional either in respect of social hierarchy, or the direction of associated effects on the PSR. To date, however, there are few studies which have directly investigated school hierarchies. One (Goodman et al., 2001), which bears close comparison with our own, involved young people ranking their family's SES position and their own position in school on a ‘ladder’, the ‘top’ referenced by students with ‘most respect, the highest grades and highest standing’, the bottom by those ‘no one respects, no one wants to hang around with and have the worst grades’. The results revealed low correlations between school position and family SES, suggesting the two are largely separate domains; further, lower school position was more strongly related to overweight and depression. While the study failed to distinguish different dimensions of school-based hierarchies, it suggests that position in the peer group may be more important than family SES for health in youth. Unfortunately, cortisol was not measured so it is not possible to directly assess the role of the PSR in the relationship.

### Cortisol

The most widely used measure of the PSR is salivary cortisol. In addition to responding to stressors, cortisol is governed by the hypothalamic–pituitary–adrenal (HPA) system and follows a daily circadian rhythm in most people. Levels are generally lowest around midnight and begin to rise before waking, thereafter rising sharply for 30–40 min as part of the cortisol awakening response (Pruessner et al., 1997). This is followed by a rapid decline for the next few hours, then a gradual decline over the remainder of the day (Kirschbaum & Hellhammer, 1989). Time of day and time of awakening, therefore, have significant effects on measured levels, which may also vary by day of the week (Maina, Palmas, & Larese Filon, 2007). Cortisol levels also vary by sex, females exhibiting higher morning cortisol (Steptoe, Cropley, Griffith, & Kirschbaum, 2000), and there is some evidence among adolescents of positive associations with age, body mass and pubertal stage (Tornhage & Alfen, 2006) and personality characteristics (Hauner et al., 2008). Over and above these variables, cortisol is responsive to a number of states and behaviours including acute illness, corticosteroid medication, eating, caffeine consumption, smoking, exercise and involvement in aggression (Kelly, Young, Sweeting, Fischer, & West, 2008). It is clear that cortisol levels respond to an individual's environment and activities, the underlying assumption being that frequent and/or sustained increases in cortisol involve negative consequences (Sapolsky, 2005).

While cortisol levels typically return to normal quite quickly after exposure to acute stressors, exposure to chronic stressors, such as those associated with lower SES position, is thought to cause dysregulation of the HPA system, typically resulting in repeatedly elevated levels (McEwen, 1998) though it may also involve particularly low levels caused by blunting of the cortisol response (Li, Power, Kelly, Kirschbaum, & Hertzman, 2007). Research on the SES/cortisol relationship is complicated by problems of capturing the diurnal rhythm, variations in the mode of collection and differences in the measures used, very few studies adequately controlling for the biological and behavioural confounds outlined above. Nevertheless, what evidence there is suggests there may be different relationships in adulthood and youth.

Among studies of adults, while a few have reported no association with SES (Decker, 2000, Dowd and Goldman, 2006) or even a positive association (Brandtstadter, Baltes-Gotz, Kirschbaum, & Hellhammer, 1991), most have found lower SES to be associated with dysregulation in some aspects of cortisol function (Cohen et al., 2006, Li et al., 2007, Ranjit et al., 2005). Among the few studies of young people, there is also some evidence of a relationship with family SES, though this tends to be based on small unrepresentative samples (Chen & Paterson, 2006; DeSantis et al., 2007). By contrast, Goodman, McEwen, Huang, Dolan, and Adler (2005) found generally weak associations with most biomarkers, and none at all with cortisol. Similarly, Lupien, King, Meaney, and McEwen (2001) found significantly elevated levels of morning cortisol in children (aged 6–10) from low compared with high SES backgrounds, but no relationship in youth (aged 12–16). Thus, while the evidence is certainly mixed, that based on more representative samples (Goodman et al., 2005, Lupien et al., 2001) appears to be consistent with the pattern of ‘relative equality’ observed for subjective health indicators in youth.

### Predictions and aims

Against this background, the overall aim of this paper is to assess the relative importance of family SES and school-based hierarchies for the PSR, as measured by a morning cortisol sample. In the absence of a strong evidence-base relating to the SES patterning of cortisol in youth, and none at all for school hierarchies, it is difficult to derive specific hypotheses. Hence, the study reported here is exploratory. However, a number of tentative predictions can be made which principally involve the distinction between more and less stable social systems and related effects on cortisol.

First, inasmuch as a young person's position in the SES hierarchy refers to a relatively stable social system, there is no a-priori reason to expect the relationship with cortisol should differ from that in adulthood. Although such evidence as there is suggests this may not be the case, it is possible the social patterning of cortisol might vary by SES measure. If social comparisons are involved as a mechanism, we might expect greater variation by FAS than either parental social class or material deprivation.

Second, while any school clearly has the potential for instability, within schools the implications for pupil ‘stress’ of formal (scholastic) and informal (peer) school hierarchies may be different. Following Sapolsky (2005), assuming the former is generally more stable, any relationship with cortisol would be an inverse one; that is, increasing levels with lower position. Conversely, the greater potential for instability in peer hierarchies suggests that levels might be elevated in higher positions.

Third, much of the work on social hierarchy and ‘stress’ assumes a generic effect, the clear prediction being a linear relationship with cortisol. An alternative view is that stress is concentrated in particular social positions, notably the bottom of a hierarchy. It is possible, however, in unstable social systems such as the peer group that this might also characterize ‘top’ positions. It is important, then, to consider the shape of the relationship between social hierarchies and cortisol.

Taking account of previously validated confounds (Kelly et al., 2008), including those of a biological and behavioural nature, we examine the relationship between cortisol and three different SES measures (parental social class, area deprivation and FAS) and three school hierarchies (scholastic, peer and sports) in univariate and mutually adjusted models to establish the extent to which any effects are independent. Controlling for confounds gives us some purchase on the mechanisms linking position in a hierarchy with cortisol. Given known sex differences in cortisol and in the makeup of the peer group, separate analyses by sex were conducted.

## Methods

### Sample and procedures

Data are derived from the ‘Peers and Levels of Stress’ (PaLS) study, a cross-sectional survey of 15-year-olds in 2006 in their final year of statutory education (Scottish S4) in 22 mainstream secondary schools situated in and around Glasgow. The sampling scheme is fully described elsewhere (Sweeting, Young, & West, 2008), but briefly involved the selection of schools within strata based on geographical location (within/outside Glasgow), religious status (Catholic/Non-Denominational) and deprivation (the proportion of pupils receiving a clothing grant), those selected being representative of secondary schools in the area. Within selected schools, all S4 pupils were invited to participate. The study received approval from Glasgow University Ethics Committee and all educational authorities, participation being achieved via opt-out parental consent together with positive consent by participants.

The survey took place in schools during the first morning class (approximately 0900 h, duration 45–55 min), representing the best compromise between the study's aim to capture the morning decline in cortisol with the practicalities of the school timetable. Pupils filled in a questionnaire, completed a brief interview, had their height and weight measured, and provided two salivary cortisol samples. Of the eligible sample of 3950 pupils, 3194 (81%) completed a questionnaire (including 137 absentees who returned their questionnaires by post). Of 3057 participants completing in school, 2995 provided two useable cortisol samples, representing 76% of the eligible sample. Missing data on confounds, together with that on one SES measure (deprivation), reduces the sample used here to 2824 (1418 males, 1406 females) with mean age 15.4 years (S.D. 0.4 years). Although the sample of schools is representative, among those completing questionnaires and providing cortisol samples there are a number of biases, including an over-representation of pupils from higher SES backgrounds. However, there were no biases according to ethnicity, the proportion self-identifying as from ‘non-white’ groups being very small (8%), reflecting the local population (Sweeting et al., 2008). Probabilistic weights were derived to compensate for bias; however, since the results of analyses using weighted and unweighted data are very similar, we present those based on the latter.

### Measures

#### Family SES

This is represented by three measures: (1) S*ocial class of the head of household*, derived from data about parental occupation obtained in the brief interview, and based on father's current occupation or, if absent or not working, mother's occupation, and coded according to the UK Registrar General's classification system (ONS, 2000). We use the full six-fold classification with the addition of a dummy variable for missing data (*n* = 278 [10%]); (2) *Area deprivation*, based on pupils' home postcodes available from school lists, these being coded by reference to 2001 Census ‘Carstairs’ scores (McLoone, 2004) and converted into standard area deprivation categories ranging from 1 (least) to 7 (most deprived); (3) *Family Affluence Scale (FAS)*, using the most recent version (Currie et al., 2008) comprising own bedroom, family car ownership, family computers and number of family holidays in the previous year, the resulting index having a range of 0–7. Here, we distinguish between low (0–3), medium (4 and 5) and high (6 and 7) FAS with an additional dummy for missing (*n* = 84 [3%]).

In common with other studies (West & Sweeting, 2004), each of the SES indicators is only moderately inter-correlated (social class/deprivation *r* = 0.35; social class/FAS *r* = −0.32; deprivation/FAS *r* = −0.34, excluding missing), supporting the view that they comprise different dimensions of family SES.

#### School-based social hierarchies

Following Goodman et al. (2001), participants were presented with seven ‘ladders’ representing a range of school-based social hierarchies; specifically popularity, academic progress, power, trouble-making, attractiveness/stylishness, respect and sportiness, each anchored with a definition of ‘top’ people (e.g. ‘most popular in your year, people who get the best grades'). Participants were asked to compare themselves with ‘the rest of the year group’ and indicate where they placed themselves by marking a cross on one of 10 rungs on each ladder, 97% (*n* = 2748) provided complete data.

The ‘ladders’ data were factor analysed (varimax), which revealed three main factors accounting for 77% of the total variance of items. The dominant factor (50% of the variance) comprised items relating to popularity, power, respect and attractiveness (each with similar high loadings) together with a smaller component of trouble-making; the second (17%) comprised two items, academic progress (the highest loading) and (not) trouble-making; the third (10%) was dominated by the single item, sportiness. Reflecting these results, the factors are labeled peer, scholastic and sports hierarchies and grouped into quintiles, the highest quintile (Q5) representing ‘top’ position in each hierarchy, those with missing data being represented by a dummy variable. Full details of the ‘ladders’, the factor analysis, and their validation are available elsewhere (Sweeting et al., submitted for publication). Analysis of the relationship between these hierarchies and family SES showed that although lower SES participants ranked themselves lower on the scholastic hierarchy, there was little or no relationship with either peer or sports hierarchies, demonstrating that school-based hierarchies are largely separate domains from family SES (Sweeting et al., submitted for publication).

#### Morning cortisol

Details of procedures for obtaining and assaying cortisol are fully described elsewhere (Kelly et al., 2008). Briefly, participants were provided with two pre-labeled Salivettes and instructed not to eat or drink during the session, and to remove chewing gum. Five minutes into questionnaire completion (T1, mean, 0917 h), the whole group was instructed to remove a cotton wool swab from the Salivette, chew on it for 2 min and replace it in the Salivette for collection by the survey team. The process was repeated half an hour later (T2) after all participants had been interviewed and had physical measures taken. Samples were stored at −20 °C and sent in batches to the laboratory for analysis. Approximately 10% of samples were run in duplicate for assessment of inter- and intra-assay reliability, those outside the detection range of the assay being repeated in appropriate dilutions, the most extreme being re-analysed in another laboratory. Extreme cases, presumed to be contaminated by blood, were excluded from analysis.

The rationale for taking two measures was that taking part in the survey (especially the physical measures) might constitute an acute stressor which would be reflected in the T2 measure. The fact that 73% of participants exhibited a decline in cortisol levels between T1 and T2 suggests this was not the case (Kelly et al., 2008). Because the time between T1 and T2 is too short to allow meaningful analysis of diurnal slopes, our preferred measure of morning cortisol is the T1 measure on the grounds that it has a greater natural range than T2 cortisol, is closer in time to behavioural confounds (see below), precedes any challenge associated with survey participation and, as the earlier measure, is a more reliable indicator of PSR (Kirschbaum et al., 1990). However, the results for T2 cortisol and for an average of the two measures are almost identical to those of T1 (details available from the authors).

#### Confounds

Since cortisol is governed by a diurnal rhythm and is affected by various states and activities, we take account of known confounds which may mediate any relationship with family SES or school hierarchies. Here, we distinguish between biological and other confounds, all fully described elsewhere (Kelly et al., 2008). Biological confounds include those directly related to the diurnal rhythm: time since awakening (the difference between self-reported awakening time and T1 cortisol), actual time of T1 cortisol, and day of the week (Monday vs. all other days) together with age (months), body mass index (BMI – ‘underweight’, ‘normal’, ‘overweight’ or ‘obese’ for age and sex) (Cole, Flegal, Nicholls, & Jackson, 2007) and interviewer rated physical maturity (below average, average and above average for age and sex). BMI is represented as a categorical rather than continuous variable because its relationship with cortisol is non-linear (Kelly et al., 2008). Other confounds include whether participants took asthma medication/used an inhaler or had a cold, together with five behaviours; whether in the previous hour they had eaten anything, drunk coffee, smoked a cigarette, exercised for more than 10 min or had a fight or argument lasting more than 20 min.

#### Analyses

Given the clustered nature of the sample, all analyses were conducted within a multilevel framework, using MLwin software (Rabash, Browne, Healy, Cameron, & Charlton, 2000) to allow for design (school) effects, though here only individual level (fixed) effects are shown The results are presented in three stages; first, adjusting only for biological confounds; second, further adjusting for all other confounds, and finally fully adjusted, i.e. for family SES and social hierarchy variables, the reference categories for both being the highest position (e.g. social class I, scholastic Q5). Dummies representing missing categories (social class, FAS and social hierarchies) are entered separately in all analyses. Because of the skewed nature of the cortisol distribution, analyses are conducted on logged T1 values. Although in general there are few significant interaction effects, those that approach or exceed conventional levels of significance involve sex; hence, results for males and females are presented separately.

## Findings

Table 1 shows the descriptive statistics of all variables used in the analysis for each sex and the total sample. Of interest is the shorter mean time (but greater variability), between awakening and T1 among males (1.80 h; mean wake time, 0729) compared with females (1.94 h; mean wake time, 0721) (*p* < 0.001). Females were assessed as more mature (*p* < 0.001) though there was no gender difference in BMI categories. A majority (64%) reported eating something in the previous hour, otherwise the proportions engaging in behaviours linked to cortisol were small, and with the exception of smoking (more females, *p* < 0.001) similar between the sexes. With respect to school hierarchies, females rated themselves higher on the scholastic hierarchy, males higher on both peer and sports hierarchies (all *p* < 0.001).

**Table 1:** Descriptive statistics of study sample with complete data.

Variable	Males (*N* = 1418)	Females (*N* = 1406)	Total (*N* = 2824)
Continuous – mean (SD)			
T1 cortisol (log)	1.027 (0.007)	1.067 (0.008)	1.047 (0.007)

Biol confounds			
Time since wakening (h)	1.80 (0.67)	1.94 (0.53)	1.87 (0.61)
Time of T1	09.28 (0.18)	09.29 (0.18)	09.29 (0.18)
Age (months)	185.61 (3.89)	185.36 (3.77)	185.49 (3.83)

Categorical – *N* (%)			
Weekday (Monday)	248 (17.5)	241 (17.1)	489 (17.3)
Phys maturity			
Below	203 (14.3)	117 (8.3)	320 (11.3)
Average	933 (65.8)	969 (68.9)	1902 (67.4)
Above	282 (19.9)	320 (22.8)	602 (21.3)
BMI Cats			
Underwt	99 (7.0)	89 (6.3)	188 (6.7)
Normal	1016 (71.7)	984 (70.0)	2000 (70.8)
Overwt	215 (15.2)	261 (18.6)	476 (16.9)
Obese	88 (6.2)	72 (5.1)	160 (5.7)

Other confounds			
Asthma medication	161 (11.4)	148 (10.5)	309 (10.9)
Cold	336 (23.7)	431 (30.7)	767 (27.2)
Eaten	919 (64.8)	881 (62.7)	1800 (63.7)
Coffee	45 (3.2)	40 (2.8)	85 (3.0)
Smoked	89 (6.3)	166 (11.8)	255 (9.0)
Exercise	205 (14.5)	200 (14.2)	405 (14.3)
Fighting	40 (2.8)	56 (4.0)	96 (3.4)

SES variables			
Social class			
I	240 (16.9)	161 (11.5)	401 (14.2)
II	396 (27.9)	377 (26.8)	773 (27.4)
IIIn	181 (12.8)	198 (14.1)	379 (13.4)
IIIm	303 (21.4)	316 (22.5)	619 (21.9)
IV	132 (9.3)	147 (10.5)	279 (9.9)
V	42 (3.0)	53 (3.8)	95 (3.4)
Missing	124 (8.7)	154 (11.0)	278 (9.8)

Deprivation			
1	117 (8.3)	115 (8.2)	232 (8.2)
2	349 (24.6)	335 (23.8)	684 (24.2)
3	141 (9.9)	110 (7.8)	251 (8.9)
4	282 (19.9)	268 (19.1)	550 (19.5)
5	255 (18.0)	285 (20.3)	540 (19.1)
6	91 (6.4)	98 (7.0)	189 (6.7)
7	183 (12.9)	195 (13.9)	378 (13.4)

FAS			
High	623 (43.9)	560 (39.8)	1183 (41.9)
Med	559 (39.4)	575 (40.9)	1134 (40.2)
Low	188 (13.3)	235 (16.7)	423 (15.0)
Missing	48 (3.4)	36 (2.6)	84 (3.0)

School hierarchies quintiles)			
Scholastic			
Q5 (top)	252 (18.3)	309 (22.6)	561 (20.4)
Q4	261 (18.9)	289 (21.1)	550 (20.0)
Q3	285 (20.7)	273 (20.0)	558 (20.3)
Q2	299 (21.7)	250 (18.3)	549 (20.0)
Q1 (btm)	283 (20.5)	247 (18.1)	530 (19.3)

Peer			
Q5 (top)	320 (23.2)	213 (15.6)	533 (19.4)
Q4	306 (22.2)	260 (19.0)	566 (20.6)
Q3	295 (21.4)	259 (18.9)	554 (20.2)
Q2	265 (19.2)	288 (21.1)	553 (20.1)
Q1 (btm)	194 (14.1)	348 (25.4)	542 (19.7)

Sports			
Q5 (top)	387 (28.0)	165 (12.1)	552 (20.1)
Q4	359 (26.0)	198 (14.5)	557 (20.3)
Q3	292 (21.2)	263 (19.2)	555 (20.2)
Q2	195 (14.1)	342 (25.0)	537 (19.5)
Q1 (btm)	147 (10.7)	400 (29.2)	547 (19.9)

Missing on hierarchies	38 (2.7)	38 (2.7)	76 (2.7)

Table 2 shows the results for the three models of T1 (logged) cortisol by family SES and school hierarchies for each sex. Focussing first on SES, the overall picture is one of little or no relationship with any measure, particularly FAS, in any model. The exception is social class in males where a slight but significant (*p* = 0.024) linear trend is observed in models adjusted first for biological and second for all confounds (largely attributable to the difference between social class I and all other classes). In the fully adjusted model, however, the trend is no longer significant. Among females, there is no significant association with social class, what little evidence of linear trends going in opposite directions for class and deprivation.

**Table 2:** Morning cortisol (mean log T1) according to family socioeconomic status (parental social class, area deprivation and family affluence) and position in school hierarchies (scholastic, peer, sports).

	Males			Females		
	Adjusted for biol confounds	Adjusted for all confounds	Fully adjusted	Adjusted for biol confounds	Adjusted for all confounds	Fully adjusted
Social class						
P (linear)	**0.024**	**0.024**	**0.161**	**0.109**	**0.161**	**0.548**
I	0.000	0.000	0.000	0.000	0.000	0.000
II	0.035*	0.036*	0.034*	0.017	0.016	0.007
IIIn	0.043*	0.040	0.038	0.027	0.023	0.012
IIIm	0.037*	0.035	0.028	0.042*	0.039	0.021
IV	0.055*	0.053*	0.049*	0.033	0.030	0.012
V	0.054	0.054	0.041	0.024	0.018	−0.007
Missing	0.018	0.018	0.001	−0.002	−0.008	−0.033

Deprivation						
P (linear)	**0.132**	**0.211**	**0.453**	**0.080**	**0.133**	**0.133**
1	0.000	0.000	0.000	0.000	0.000	0.000
2	−0.018	−0.024	−0.029	−0.023	−0.023	−0.027
3	−0.013	−0.021	−0.023	−0.002	−0.002	−0.010
4	0.020	0.013	0.003	0.013	0.009	−0.005
5	−0.010	−0.018	−0.025	−0.006	−0.010	−0.022
6	0.034	0.023	0.015	0.000	−0.002	−0.010
7	0.022	0.013	0.003	0.036	0.032	0.029

FAS						
P (linear)	**0.579**	**0.826**	**0.912**	**0.502**	**0.502**	**0.742**
High	0.000	0.000	0.000	0.000	0.000	0.000
Med	0.004	0.002	−0.001	0.025	0.023	0.020
Low	0.011	0.005	0.003	0.004	0.003	−0.004
Missing	0.022	0.020	0.015	0.026	0.018	−0.006

Scholastic						
P (linear)	**0.012**	**0.080**	**0.133**	**0.001**	**0.006**	**0.006**
Q5 (Top)	0.000	0.000	0.000	0.000	0.000	0.000
Q4	0.011	0.010	0.009	0.013	0.013	0.007
Q3	0.024	0.020	0.019	0.042*	0.041*	0.036*
Q2	0.021	0.016	0.017	0.032	0.030	0.024
Q1 (Btm)	0.043*	0.034	0.029	0.053**	0.045*	0.040*

Peer						
P (linear)	**0.006**	**0.012**	**0.006**	**0.317**	**0.452**	**0.617**
Q5 (Top)	0.000	0.000	0.000	0.000	0.000	0.000
Q4	−0.017	−0.014	−0.011	−0.042*	−0.038*	−0.034
Q3	−0.017	−0.016	−0.015	−0.028	−0.023	−0.017
Q2	−0.033*	−0.029	−0.028	−0.053**	−0.046*	−0.040*
Q1 (Btm)	−0.047*	−0.043*	−0.047*	−0.024	−0.017	−0.014

Sports						
P (linear)	**0.045**	**0.211**	**0.133**	**0.002**	**0.006**	**0.012**
Q5 (Top)	0.000	0.000	0.000	0.000	0.000	0.000
Q4	0.002	0.000	−0.007	0.047*	0.046*	0.042
Q3	0.019	0.015	0.009	0.033	0.030	0.019
Q2	−0.007	−0.015	−0.015	0.059**	0.056**	0.049*
Q1 (Btm)	0.049*	0.038	0.047*	0.057**	0.053**	0.048*

Interactions (*p* value) sex*SES/hierarchies in 3 models (biol; all; fully adjusted): social class (0.992; 0.992; 0.992), deprivation (0.454; 0.454; 0.689), FAS (0.841; 0.764; 0.586), scholastic (0.231; 0.318; 0.502), peer (0.045; 0.027; 0.067), sports (0.095; 0.067; 0.242). All results adjusted (1) for biological confounds (time since awakening, time of first cortisol measure and weekday [Monday/rest], maturity and BMI) (2) all (biological + other) confounds (asthma med, cold eaten, coffee, smoked, exercise and fighting) (3) fully adjusted for biological and behavioural confounds and SES and social hierarchies. *P* for linear trend indicated in bold; sig deviations from ref category *p < 0.05; **p < 0.01; ***p < 0.001.

The lack of relationship between SES and cortisol contrasts with that for school-based hierarchies. For the scholastic hierarchy, adjusting only for biological confounds, there is a significant inverse gradient in both sexes, cortisol increasing with lower position and being particularly elevated in those at the ‘bottom’ (Q1). In males, this trend becomes non-significant when further adjusted for all other confounds (analysis shows this to be largely due to higher levels of eating and smoking among those in lower positions) while in females the effect is less marked. In the fully adjusted model, the significant linear gradient (*p* = 0.006) for females remains.

With respect to the other hierarchies, a different relationship is observed, which also varies by sex. For peer hierarchy, among males there is a direct linear relationship, which is barely affected by progressive adjustment, the full model showing cortisol decreasing with each quintile (*p* = 0.006). Among females, there is no evidence of a gradient in any model; rather all positions except the ‘top’ (Q5) have lower cortisol. For sports hierarchy, among males although there is some evidence of a gradient in the first model, the clearest pattern throughout is that which distinguishes those at the ‘bottom’ as having higher cortisol. Among females, evidence of a persistent gradient obscures the fact that the major difference is between ‘top’ position and all lower positions, those with highest status having lower cortisol.

In general, relationships between social hierarchies and cortisol are only marginally affected by adjustment for biological and other (mainly behavioural) confounds. Most importantly, the fully adjusted model demonstrates the independence of their effects both within school and with respect to family SES. The different patterns for peer and sports hierarchies between the sexes are also reflected in significant or near significant interaction terms in models conducted on the total sample (see Table 2 footnotes).

## Discussion

Although there are many studies investigating family SES and the peer group as separate domains, very few examine their effects together. Our findings provide new evidence on the issue, demonstrating different associations between family SES and school-based social hierarchies and the PSR, as represented by cortisol. The overall conclusion is that school hierarchies are a more important source of ‘stress’ at this age than family SES, however, measured. The strength of this conclusion is enhanced by the finding of independent effects of three distinct school hierarchies, each with a different relationship to cortisol, standing in contrast to, and validating, the finding of little or no SES variation.

From a life-course perspective (Li et al., 2007), the lack of relationship between family SES and cortisol in youth is unexpected since the assumption is that stressors accumulate from birth onwards. While a glimpse of this may be discernible in the social class trend in males, this should be set alongside the negative findings for two other measures (deprivation and FAS). In a sub-analysis of the extremes of cortisol (bottom 10%), we failed to find any evidence that low SES was associated with blunting of the cortisol response (details available from the authors). From a social comparisons perspective (Wilkinson, 1996), inasmuch as the effect of negative comparisons on the PSR is best indicated by FAS, there is also no support for the hypothesis. Our finding of little or no SES patterning of cortisol is consistent both with two other studies using representative samples of young people (Goodman et al., 2005, Lupien et al., 2001), and most of the evidence relating to health in youth (West, 1997).

Following Sapolsky (2005), an important distinction exists between the expected effects of ‘stress’ in stable vs. unstable social systems. The identification of three distinct school-based hierarchies suggests the possibility that each might be associated with cortisol in different ways. With respect to the official (stable) school system, the expectation is that hierarchical position would be inversely related to cortisol; that is, higher cortisol with lower status. The results for the scholastic hierarchy are consistent with this prediction, particularly in females. This in turn is consistent with evidence that academic success is more salient, and potentially more ‘stressful’, for females (West & Sweeting, 2003). Among males, one interpretation of the reduced effect when other confounds are added is that ‘stress’ associated with lower positions is mediated by behaviours like smoking and eating. An alternative explanation is selection; rather than position in the scholastic hierarchy bestowing ‘stress’, individuals with different PSRs are selected, or select themselves, for social position. Given that the two key components of the scholastic factor are ‘doing well at school’ and (not) being a ‘trouble-maker’, evidence that cortisol levels may be related to early cognitive ability (Wiedenmayer et al., 2006) and anti-social behaviour in adolescence (Popma et al., 2007), is compatible with this explanation. However, it is unlikely this is a complete explanation, not least because it does not easily fit with the findings for either the peer or sports hierarchies.

Given the potential for instability associated with peer hierarchies, the expectation deriving from Sapolsky's work, is that the relationship with cortisol might be reversed; higher levels being associated with higher status. The results for peer hierarchy are only partially consistent with the prediction. While in males there is a clear gradient, in females it is those at the ‘top’ who are distinguished from other positions as having higher cortisol. Why this might be is uncertain, but it could be that competition for status among males is experienced at all levels while for females it is stressors associated with the position of ‘top girl’ that matter (Michell & Amos, 1997).

Arguably, the sports hierarchy occupies mid position between scholastic and peer hierarchies. As with peer hierarchy, our results are only partially consistent with predictions, neither sex exhibiting a clear gradient. However, the relationship differs between them. In males, only those at the bottom of the hierarchy have elevated cortisol; in females it is all those except the ‘top’ who have higher levels. Interpretation is difficult, the absence of a gradient suggesting it does not reflect the official school system in a similar way to the scholastic hierarchy. It seems more likely it is interpretable as another dimension of peer status which strongly distinguishes the sexes. Thus, given the importance of athletic prowess for males, it may be that doing something is better than doing nothing, only those in the lowest position experiencing negative reactions and associated ‘stress’. For females, doing little or nothing may matter much less, only those at the ‘top’ being distinguished as different, their lower cortisol possibly being a direct result of higher levels of physical activity. An alternative explanation in terms of selection seems less likely given that the results control for factors relating to fitness, notably BMI.

Understanding these relationships between school-based hierarchies and cortisol requires further investigation of the processes by which status is allocated. Unfortunately, no data were collected on the salience of scholastic, peer and sports dimensions, which would have aided interpretation of the different patterns for males and females. It is clear though, that the three hierarchies are separate from one another and that the effects on cortisol are hierarchy specific. Thus, the cumulative effect of different positions in these social hierarchies may reduce or elevate cortisol, but they may also cancel each other out. For example, being at the top of the scholastic, but the bottom of the peer hierarchy may be protective. Focussing on the extremes of social hierarchies, however, detracts from a key finding, which is that for the scholastic hierarchy in both sexes, and peer hierarchy in males, the relationship with cortisol takes the form of a gradient. As with evidence relating to the SES patterning of health in adulthood (Marmot, 2004), this suggests that the mechanisms involved are intrinsically related to social hierarchy, affecting individuals at all points within it. Although the FAS results provide no support for a social comparisons perspective, this may be too crude an indicator of what matters to young people. In the microcosm of the school, with the twin challenges of competitive scholastic goals and achieving valued youth identities, it is more likely social comparisons are a feature of everyday life.

There are several caveats to these conclusions arising from limitations of the study. First, and most important, the measures of cortisol obtained in ‘PaLS’ were necessarily restricted by the school timetable. Ideally, it would have been desirable to get a fuller picture of participants' diurnal rhythms, particularly the cortisol awakening response since there is evidence it is regulated by a distinct mechanism (Clow, Thorn, Evans, & Hucklebridge, 2004). The absence of such data, and the collection of cortisol at a standard time (T1) means it is not possible to distinguish individuals with different post-awakening cortisol trajectories. How this might affect results is unknown; the two most comparable studies (Goodman et al., 2005, Lupien et al., 2001) also collected data on morning (not awakening) response. The reasons for our decision to present, and, therefore, prioritise, results based on the first (T1) of two measures have been given earlier; in summary, we believe it to be the more reliable indicator of PSR in this study.

Second, following Sapolsky, the underlying assumption in our analyses is that exposure to stressors results in elevated cortisol levels. However, this is not the only possible indicator of PSR dysregulation, recent studies emphasizing the importance of very low levels following exposure to chronic stressors (Li et al., 2007). In justification of our assumption that elevated cortisol is more typically associated with negative effects, it is worth noting the association with two confounds, smoking and obesity, both associated with poorer health. While this cannot be assumed to involve a causal relationship, it is consistent with the literature showing elevated cortisol in individuals with several chronic health conditions (e.g. Decker et al., 2008).

Third, while our analyses provide no support for the postulated blunted cortisol response associated with low SES, it is important to acknowledge another limitation relating to the sample. Although the weighting procedure developed for the study corrects for known biases, we cannot rule out the possibility that young people most exposed to chronic stressors were excluded from the study.

Fourth, the study is cross-sectional, ruling out any consideration of the impact of changing social position on cortisol. This is particularly important in relation to peer hierarchy, since predictions about the direction of the effect on cortisol are premised on the notion that peer group processes are dynamic, and that it represents an unstable social system. It also limits conclusions about the direction of causality between school hierarchies and cortisol, in particular making it difficult to evaluate the role of selection. The absence of data on cognitive ability and personality are particularly important in this respect. However, the specificity of the results for each of the social hierarchies would not be expected if selection were the major explanation. In addition, controlling for confounds, notably BMI in relation to the sports hierarchy, suggests an effect of social position on the PSR rather than the reverse.

Finally, underpinning the analysis is the assumption that the measures used in ‘PaLS’, which refer to subjective social status, are good measures of objective social hierarchies with real effects on ‘stress’. In a related paper (Sweeting et al., submitted for publication), we have demonstrated relationships between position in these hierarchies and a range of attributes which appear to validate their more objective status. For example, there are strong relationships between scholastic position and number and grade of exams entered, between peer status and experience of victimization, and between sports status and obesity. Nevertheless, hierarchical position is derived from perceived position on a number of ‘ladders’ and it remains to be demonstrated how good young people are at knowing their place in these hierarchies.

Notwithstanding these caveats, the findings strongly support the conclusion that young people's school-based peer groups are largely separate domains of influence from their social background; that there is not a single school hierarchy but multiple hierarchies; that each is more important for PSR than family SES; and that the manner in which this plays out as an effect on cortisol depends both on the position occupied, and in which hierarchy.

## References

[bib1] Brandtstadter J., Baltes-Gotz B., Kirschbaum C., Hellhammer D. (1991). Development and personality correlates of adrenocortical activity as indexed by salivary cortisol: observations in the age range 35 to 65 years. Journal of Psychosomatic Research.

[bib2] Chen E., Paterson L.Q. (2006). Neighbourhood, family, and subjective socioeconomic status: how do they relate to adolescent health?. Health Psychology.

[bib3] Cillessen A.H.N., Rose A.J. (2005). Understanding popularity in the peer system. Current Directions in Psychological Science.

[bib4] Clow A., Thorn L., Evans P., Hucklebridge F. (2004). The awakening cortisol response: methodological issues and significance. Stress.

[bib5] Cohen S., Schwartz J.E., Epel E., Kirschbaum C., Sidney S., Seeman T. (2006). Socioeconomic status, race, and diurnal cortisol decline in the Coronary Artery Risk Development in Young Adults (CARDIA) study. Psychosomatic Medicine.

[bib6] Cole T., Flegal K., Nicholls D., Jackson A. (2007). Body mass index cutoffs to define thinness in children and adolescents: international survey. British Medical Journal.

[bib7] Currie C., Molcho M., Boyce W., Holstein B., Torsheim T., Richter M. (2008). Researching health inequalities in adolescents: the development of the Health Behaviour in School-Aged Children (HBSC) family affluence scale. Social Science & Medicine.

[bib8] Decker M.J.H.J., Koper J.W., van Aken M.O., Pols H.A.P., Hofman A., de Jong F.H. (2008). Salivary cortisol is related to atherosclerosis of carotid arteries. Journal of Clinical Endocrinology & Metabolism.

[bib9] Decker S.A. (2000). Salivary cortisol and social status among Dominican men. Hormones and Behavior.

[bib10] Demakakos P., Nazroo J., Breeze E., Marmot M. (2008). Socioeconomic status and health: the role of subjective social status. Social Science & Medicine.

[bib11] DeSantis A.S., Adam E.K., Doane L.D., Mineka S., Zinberg R.E., Craske M.G. (2007). Racial/ethnic differences in cortisol diurnal rhythms in a community sample of adolescents. Journal of Adolescent Health.

[bib12] Dowd J.B., Goldman N. (2006). Do biomarkers of stress mediate the relation between socioeconomic status and health?. Journal of Epidemiology and Community Health.

[bib13] Giordano P. (2003). Relationships in adolescence. Annual Review of Sociology.

[bib14] Goodman E., Adler N.E., Kawachi I., Frazier A.L., Huang B., Colditz G.A. (2001). Adolescents' perceptions of social status: development and evaluation of a new indicator. Pediatrics.

[bib15] Goodman E., McEwen B.S., Huang B., Dolan L., Adler N.E. (2005). Social inequalities in biomarkers of cardiovascular risk in adolescence. Psychosomatic Medicine.

[bib16] Hauner K., Adam E., Mineka S., Doane L.D., DeSantis A.S., Zinbarg R. (2008). Neuroticism and introversion are associated with salivary cortisol patterns in adolescents. Psychoneuroendocrinology.

[bib17] Holstein B., Parry-Langdon N., Zambon A., Currie C., Roberts C., Currie C. (2004). Socioeconomic inequality and health. Young people's health in context: International report from the HBSC 2001/02 survey.

[bib18] Kelly S.J., Young R., Sweeting H., Fischer J.E., West P. (2008). Levels and confounders of morning cortisol collected from adolescents in a naturalistic (school) setting. Psychoneuroendocrynology.

[bib19] Kirschbaum C., Hellhammer D.H. (1989). Salivary cortisol in psychobiological research: an overview. Neuropsychobiology.

[bib20] Kirschbaum C., Steyer R., Eid M., Patalla U., Schwenkmezger P., Hellhammer D.H. (1990). Cortisol and behavior: 2. Application of a latent-state-trait model to salivary cortisol. Psychoneuroendocrinology.

[bib21] Kunz-Ebrecht S.R., Kirschbaum C., Steptoe A. (2004). Work stress, socioeconomic status and neuroendocrine activation over the working day. Social Science & Medicine.

[bib22] Li L., Power C., Kelly S., Kirschbaum C., Hertzman C. (2007). Lifetime socioeconomic position and cortisol patterning in mid-life. Psychoneuroendocrynology.

[bib23] Lupien S.J., King S., Meaney M.J., McEwen B.S. (2001). Can poverty get under your skin? Basal cortisol levels and cognitive function in children from low to high socioeconomic status. Development and Psychpathology.

[bib24] Macintyre S. (1997). The Black report and beyond: what are the issues?. Social Science & Medicine.

[bib25] Maina G., Palmas A., Larese Filon F. (2007). Relationship between self-reported mental stressors at the workplace and salivary cortisol. International Archive of Occupational & Environmental Health.

[bib26] Marmot M. (2004). Status syndrome: How your social standing directly affects your health and life expectancy.

[bib27] McEwen B.S. (1998). Protective and damaging effects of stress mediators. New England Journal of Medicine.

[bib28] McLoone P. (2004). Carstairs scores for Scottish postcode sectors from the 2001 census.

[bib29] Meisinger E., Blake J., Lease A., Palardy G., Olejnik S. (2007). Variant and invariant predictors of perceived popularity across majority-Black and majority-White classrooms. Journal of School Psychology.

[bib30] Michell L., Amos A. (1997). Girls, pecking order and smoking. Social Science & Medicine.

[bib31] Milner M. (2006). Freaks, geeks and cool kids.

[bib32] ONS (2000). Standard occupational classification.

[bib33] Popma A., Doreleijers T., Jansen L., Van Goozen H., Van Engeland H., Verneiren R. (2007). The diurnal cortisol cycle in delinquent male adolescents and normal controls. Neuropsychopharmacology.

[bib34] Pruessner J.C., Wolf O.T., Hellhammer D.H., Buske-Kirschbaum A., Van Auer K., Jobst S. (1997). Free cortisol levels after awakening: a reliable biological marker for the assessment of adrenocortical activity. Life Sciences.

[bib35] Rabash J.W., Browne W., Healy M., Cameron B., Charlton C. (2000). MlwiN version 1.10.

[bib36] Ranjit N., Young E.A., Kaplan G.A. (2005). Material hardship alters the diurnal rhythm of salivary cortisol. International Journal of Epidemiology.

[bib37] Sapolsky R.M. (2005). The influence of social hierarchy on primate health. Science.

[bib38] Steptoe A., Cropley M., Griffith J., Kirschbaum C. (2000). Job strain and anger expression predict early morning elevations in salivary cortisol. Psychosomatic Medicine.

[bib39] Sweeting, H., West, P., Young, R., & Kelly, S. Dimensions of social hiearchy among young people: description and validation. *Journal of Adolescence*, submitted for publication.

[bib40] Sweeting H., Young R., West P. (2008). The Peers and Levels of Stress (PaLS) Study; basic frequencies and documentation.

[bib41] Tornhage C.-J., Alfen G. (2006). Diurnal salivary cortisol concentration in school-aged children: increased morning cortisol concentration and total cortisol concentration negatively correlated to body mass index in children with recurrent abdominal pain of psychosomatic origin. Journal of Pediatric Encrinology & Metabolism.

[bib42] West P. (1997). Health inequalities in the early years: is there equalisation in youth?. Social Science & Medicine.

[bib43] West P., Sweeting H. (2003). Fifteen, female and stressed: changing patterns of psychological distress over time. Journal of Child Psychology & Psychiatry.

[bib44] West P., Sweeting H. (2004). Evidence on equalisation in health in youth from the West of Scotland. Social Science & Medicine.

[bib45] Wiedenmayer C., Bansal R., Anderson G., Zhu H., Arnat J., Peterson B. (2006). Cortisol levels and hippocampus volumes in healthy preadolescent children. Psychiatry.

[bib46] Wilkinson R.G. (1996). Unhealthy societies: The affliction of inequality.

